# Reorganization of the Brain Structural Covariance Network in Ischemic Moyamoya Disease Revealed by Graph Theoretical Analysis

**DOI:** 10.3389/fnagi.2022.788661

**Published:** 2022-06-02

**Authors:** Peijing Wang, Wenjie Li, Huan Zhu, Xingju Liu, Tao Yu, Dong Zhang, Yan Zhang

**Affiliations:** ^1^Department of Neurosurgery, Beijing Tiantan Hospital, Capital Medical University, Beijing, China; ^2^China National Clinical Research Center for Neurological Diseases, Beijing, China; ^3^Center of Stroke, Beijing Institute for Brain Disorders, Beijing, China; ^4^Beijing Key Laboratory of Translational Medicine for Cerebrovascular Disease, Beijing, China

**Keywords:** moyamoya disease, structural covariance network, graph theory, cerebral gray matter, voxel-based morphometry

## Abstract

**Objective:**

Ischemic moyamoya (MMD) disease could alter the cerebral structure, but little is known about the topological organization of the structural covariance network (SCN). This study employed structural magnetic resonance imaging and graph theory to evaluate SCN reorganization in ischemic MMD patients.

**Method:**

Forty-nine stroke-free ischemic MMD patients and 49 well-matched healthy controls (HCs) were examined by T1-MPRAGE imaging. Structural images were pre-processed using the Computational Anatomy Toolbox 12 (CAT 12) based on the diffeomorphic anatomical registration through exponentiated lie (DARTEL) algorithm and both the global and regional SCN parameters were calculated and compared using the Graph Analysis Toolbox (GAT).

**Results:**

Most of the important metrics of global network organization, including characteristic path length (Lp), clustering coefficient (Cp), assortativity, local efficiency, and transitivity, were significantly reduced in MMD patients compared with HCs. In addition, the regional betweenness centrality (BC) values of the bilateral medial orbitofrontal cortices were significantly lower in MMD patients than in HCs after false discovery rate (FDR) correction for multiple comparisons. The BC was also reduced in the left medial superior frontal gyrus and hippocampus, and increased in the bilateral middle cingulate gyri of patients, but these differences were not significant after FDR correlation. No differences in network resilience were detected by targeted attack analysis or random failure analysis.

**Conclusions:**

Both global and regional properties of the SCN are altered in MMD, even in the absence of major stroke or hemorrhagic damage. Patients exhibit a less optimal and more randomized SCN than HCs, and the nodal BC of the bilateral medial orbitofrontal cortices is severely reduced. These changes may account for the cognitive impairments in MMD patients.

## Introduction

Moyamoya disease (MMD) is a chronic cerebrovascular disorder characterized by the progressive occlusion of terminal internal carotid arteries and/or other large intracranial arteries, resulting in the formation of collateral artery networks that manifest as “puffs of smoke” on digital subtraction angiography (DSA) (Suzuki and Takaku, 1969; Kuroda and Houkin, 2008; Scott and Smith, 2009). The heterogeneous loci of these ischemic events can result in a variety of distinct clinical symptoms, and these often include various cognitive impairments that can increase the difficulty of independent living (Festa et al., 2010; Gorelick et al., 2011). Moreover, MMD can impair cognition even before detectable ischemic events (Kazumata et al., 2015; Li et al., 2019). The mechanisms contributing to these deficits remain to be understood.

Over the last two decades, a multidisciplinary approach, known as complex network analysis, has been applied to demonstrate the important properties of connecting patterns among these brain regions based on graph theory. In this approach, nodes represent brain regions, while edges/connections among nodes are defined by temporal correlations on functional magnetic resonance imaging (functional MRI, fMRI), morphological correlations on structural MRI (sMRI), or tracing fibers on diffusion tensor imaging (DTI) (Friston, 1994; Rubinov and Sporns, 2010). Individual brain regions are connected according to network topology rules that ideally optimize “small-worldness,” a network property that maintains optimal balance between local processing and global interaction, thereby facilitating rapid synchronization and efficient information transfer while minimizing wiring costs (Stam and Reijneveld, 2007; Rubinov and Sporns, 2010). Maintaining this specific network organization is crucial for higher-level cognitive function requiring the integration of multimodal information and can be altered by neural system diseases (Achard and Bullmore, 2007). Graphical analysis of brain structural covariance networks (SCNs) (networks constructed based on statistical correlations of the morphological indices among cerebral regions) can provide comprehensive information at network level and provide clues to neuropathological mechanisms. However, previous brain morphological analyses of MMD mainly focused on the changes in cortical volume/thickness and characteristics of atrophy (Qiao et al., 2017; Su et al., 2019), leaving SCN unexplored.

In this study, we examined SCN changes in ischemic MMD, focusing on patients without ischemic or hemorrhagic stroke because the resulting cortical hemosiderosis and tissue damage may complicate image analysis. We hypothesized that ischemic MMD could alter and reorganize the intrinsic properties of SCN, and that the disturbed connectivity among cortical regions may be a potential mechanism of cognitive impairments in MMD patients. To test these postulates, we used voxel-based morphometry (VBM) based on diffeomorphic anatomical registration through exponentiated lie (DARTEL) algorithm to obtain precise gray matter (GM) images of ischemic MMD patients and matched controls, and then used the Graph Analysis Toolbox (GAT) (Hosseini et al., 2012) to construct SCNs and identify differences in topological properties specific to MMD.

## Materials and Methods

### Participants

This study was approved by the ethics committee of Beijing Tiantan Hospital, Capital Medical University. All MMD patients and healthy control participants were volunteers and provided informed consent. Basic demographic information, such as age, sex, and educational background, was obtained by interview. The Suzuki stages (Suzuki and Takaku, 1969) and the Fazekas scales (Fazekas et al., 1987) were quantified by radiologists based on DSA/MRA and FLAIR images, respectively. From November 2018 to January 2021, 49 stroke-free ischemic MMD patients were enrolled according to the following inclusion criteria: (1) diagnosed with bilateral MMD according to the criteria of the Research Committee on Spontaneous Occlusion of the Circle of Willis; (2) over 18 years of age; (3) no evidence of intracerebral hemorrhage or infarct larger than 8 mm on structural images; (4) no cranial surgery prior to recruitment; (5) no history of any other cognitive impairment diseases or drug use that may alter cognitive function; (6) no MRI contraindications. Forty-nine healthy controls (HCs) strictly matched for age, sex, and educational background were recruited using the following criteria: (1) no history of neurological, psychiatric, or cognitive diseases; (2) no history of drug use that could alter cognitive function; (3) no MRI contraindications.

### Magnetic Resonance Imaging Acquisition

Structural brain images were acquired at Beijing Tiantan Hospital using an Ingenia 3.0 Tesla scanner (Philip Medical Systems, Best, Netherlands) equipped with a 32-channel head coil. A T1-weighted MPRAGE sequence with the following parameters was used for all scans: TR 6.84 ms, TE 3.09 ms, flip angle 8°, FOV 240 × 240 mm^2^, matrix 240×240, slice thickness 1.0 mm and voxel size 1.0 × 1.0 × 1.0 mm^3^.

### Magnetic Resonance Imaging Image Processing

T1-MPRAGE images were processed automatically using the Computational Anatomy Toolbox 12 (CAT12) extension of Statistical Parametric Mapping 12 (SPM12) running in MATLAB (2018b, MathWorks, Natick, MA, United States). Image processing steps included bias field correction, skull dissection, alignment with the Montreal Neurological Institute standard space (MNI-152 template), and segmentation into GM, white matter (WM), and cerebrospinal fluid (CSF). A group-specific template was generated using the DARTEL algorithm (Ashburner, 2007). Segmented images in native space were then subjected to non-linear warping and normalized to match the DARTEL templates. During this procedure, images were modulated to ensure preservation of relative GM and WM volumes. Finally, the modulated and normalized images were smoothed using an 8-mm full-width at half-maximum isotropic Gaussian kernel.

### Structural Covariance Network Construction

Structural covariance networks were constructed using the Graph Analysis Toolbox (GAT) (Hosseini et al., 2012). Images were first parcellated using the Automated Anatomical Labelling (AAL) template, and the 90 cortical and subcortical regions defined by AAL were set as regions of interest (ROIs) for VBM. For each group, regional GM volumes of the 90 ROIs were extracted and a 90×90 association matrix was constructed by calculating Pearson’s correlation coefficients between all ROI volumes. Total intracranial volume (TIV) was set as a nuisance covariate and its influence was removed by linear regression. The minimum edge density was set at 0.27 (Dmin = 0.27) to ensure that the SCNs of both groups were fully connected, while the maximum density was set at 0.5 as greater density is considered non-biological (Kaiser and Hilgetag, 2006; Hosseini et al., 2012). The SCN binary matrix was then thresholded within this range of densities (from 0.27 to 0.5 at an interval of 0.01).

Two crucial network metrics, the clustering coefficient (Cp) and the characteristic path length (Lp), were then calculated. The Cp is a measure of functional segmentation obtained by first counting the Cp of each node—the ratio of the existing edges between its neighboring nodes to the maximal possible number of edges between them—and then calculating the average across all nodes (Watts and Strogatz, 1998; Rubinov and Sporns, 2010). The Lp is the most commonly used measure of functional integration and is calculated as the average of the shortest path length between all pairs of nodes in the network (Watts and Strogatz, 1998; Rubinov and Sporns, 2010). These two metrics were then divided by the Cp and Lp values of 20 random null networks, termed Cprand and Lprand, respectively, to obtain the normalized Cp (γ = Cp/Cprand) and normalized Lp (λ = Lp/Lprand). The small-world index (σ = γ/λ) was then calculated (Watts and Strogatz, 1998). A brain network with γ > 1, and λ ≈ 1 or σ > 1, is deemed to possess small-world properties (Rubinov and Sporns, 2010).

For global SCN analysis, the following additional network metrics were also calculated: global efficiency, local efficiency, assortativity, transitivity, and modularity. Global efficiency is the average of the inverse of the shortest path length between all nodes in the network (Latora and Marchiori, 2001). Local efficiency is the average of the inverse of shortest path length between adjacent nodes of each given node (Latora and Marchiori, 2001; Vragovic et al., 2005). Assortativity reflects the likelihood of node attachment to other network nodes with the same degree (Newman, 2002), while transitivity, a variant of the clustering coefficient, is a measure of network segregation (Newman and Park, 2003). Modularity reflects the degree to which the whole network can be divided into cliques, where a clique is a cluster of densely interconnected nodes that are less well-connected to other nodes and clusters (Girvan and Newman, 2002; Newman, 2006). Then, for regional SCN analysis, we focused mainly on the normalized nodal betweenness coefficient (BC), defined as the fraction of all shortest paths passing through a given node normalized to the average BC of the entire network (Freeman, 1977), as this metric reflects the importance of a given node in controlling information flow. Finally, network resilience reflects the tolerance to random failure and targeted attack. It is measured by the change in the relative size of the remaining connected components after removing individual nodes randomly or in descending order of BC until all nodes are removed (Achard et al., 2006).

### Statistical Analysis

All basic variables (age, sex, and educational level) were compared between groups using SPSS (Windows version 23.0, IBM). Continuous variables are expressed as mean ± standard deviation and categorical values as numbers (percentages). Categorical variables were compared between groups by the chi-square test, continuous variables by the Student’s *t*-test and ranked data by the rank-sum test. A *P* < 0.05 was considered significant for all SPSS tests.

Network measures were compared between groups using GAT. Non-parametric permutation tests, each with 1,000 repetitions, were performed to test the statistical significance of between-group differences in global and regional network measures, with *P* < 0.05 (two-tailed) considered significant. Permutation analysis was also performed to assess between-group differences in network resilience against random failure and targeted attack, with statistical significance set at *P* < 0.05. To reduce the impact of thresholding, we also compared the areas under the curves (AUCs) generated from density variation between groups. In addition, the false discovery rate (FDR) was applied to correct for multiple comparisons in the regional BC analysis, with *P* < 0.05 (FDR-corrected) considered statistically significant.

## Results

### Demographics and Group Matching

Table 1 summarizes the baseline demographic data of MMD patients and HCs. Two groups were well-matched for age, sex ratio, and educational background (all *P* > 0.05).

**TABLE 1:** Basic information of MMD patients and healthy controls.

	MMD patients	Healthy controls	*P*-value
Number	49	49	–
Age/years	44.67 ± 11.06	44.67 ± 11.06	1.000
Sex/male	22 (44.9%)	22 (44.9%)	1.000
TIV	1471.76 ± 129.68	1483.26 ± 118.10	0.459
**Educational level**			1.000
Primary school	3 (6.1%)	3 (6.1%)	
Junior high school	15 (30.6%)	15 (30.6%)	
Senior high school	15 (30.6%)	15 (30.6%)	
College/above	16 (32.7%)	16 (32.7%)	
**Symptoms**			–
TIA	33 (67.3%)	–	
Headache/dizziness	15 (30.6%)	–	
Non-symptom	1 (2.1%)		
**Suzuki stage (left/right)**			–
I	2 (4.1%)/2 (4.1%)	–	
II	1 (2.0%)/0 (0%)	–	
III	32 (65.3%)/34 (69.4%)	–	
IV	14 (28.6%)/13 (26.5%)	–	
**Fazekas scale**			–
0	13 (26.5%)	–	
1	30 (61.2%)	–	
2	6 (12.3%)	–	
3	0 (0%)	–	
Lacunar infarction	30 (61.2%)	–	–

*TIV, total intracranial volume.*

### Global Structural Covariance Network Measures

**FIGURE 1:**
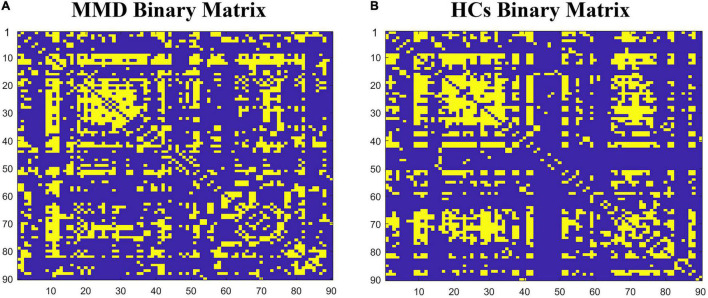
The binary matrices of patients with Ischemic MMD patients **(A)** and healthy controls **(B)** at Dmin. The X/Y axes represent the 90 cerebral regions from the AAL atlas and the specific order of regions is listed in Supplementary Table 1.

**FIGURE 2:**
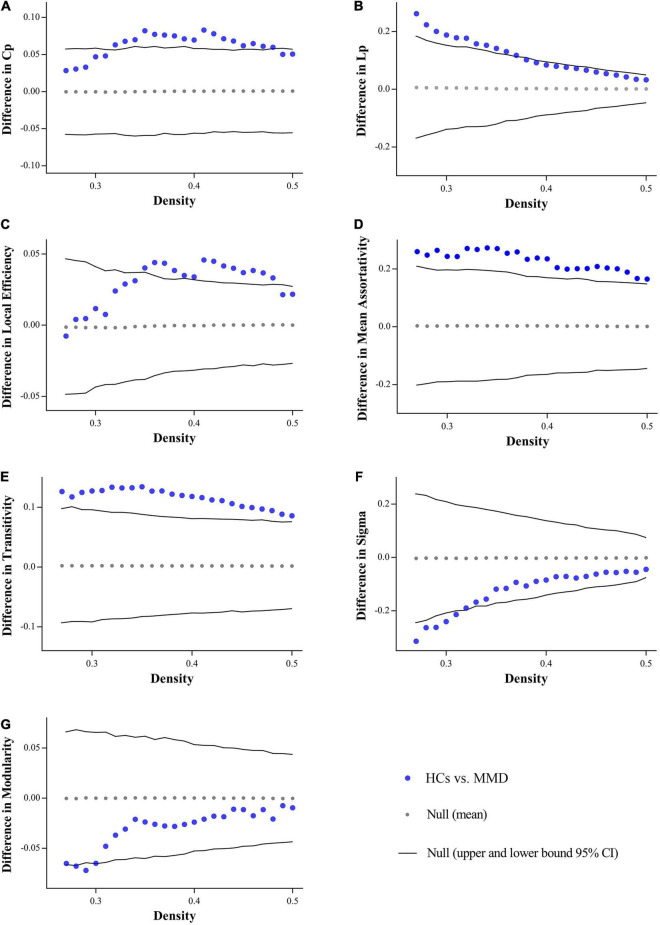
Between-group differences in global measures across density range. MMD patients exhibit significantly lower clustering coefficient (Cp) **(A)**, characteristic path length (Lp) **(B)**, local efficiency **(C)**, assortativity **(D)**, transitivity **(E)** at several densities across the range, while showing significantly higher Sigma **(F)**, and modularity **(G)** at some density points. Cp, clustering coefficient; Lp, characteristic path length.

The binary matrices of two groups are shown in Figure 1. The SCNs in both groups exhibited small-worldness as indicated by all γ > 1 with λ ≈ 1 or σ > 1 across the density range (0.27:0.01:0.5). The small-word indices were significantly higher in the MMD group than in the control group at several points along the density range (0.27:0.01:0.5) (all *P* < 0.05). Compared to the SCNs of HCs, the SCNs of MMD patients also exhibited significantly lower Lp, Cp, assortativity, local efficiency, and transitivity values at several densities across the range (all *P* < 0.05) (Figure 2).

In addition to comparing SCN measures at each density across the range, we also compared the AUCs for global SCN measures between groups across the density range (0.27:0.01:0.5). Consistent with the above results, the AUC comparisons indicated significantly lower Lp (*P* = 0.030), Cp (*P* = 0.023), assortativity (*P* = 0.009), local efficiency (*P* = 0.023), and transitivity (*P* = 0.009) in MMD group, whereas modularity (*P* = 0.211) and small-world index (*P* = 0.100) did not reach the statistical significance.

### Regional Betweenness Centrality

**FIGURE 3:**
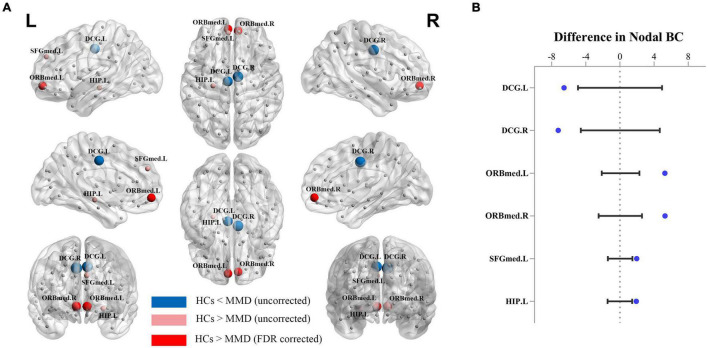
Between-group differences in reginal betweenness centrality (BC). **(A)** 3D images. **(B)** Data of between-group differences. Red color identifies the regions with significantly higher BC in MMD without false discovery rate (FDR) correction, while red and pink color identify regions with significantly higher BC in HCs with and without FDR correction, respectively. DCG.L, left middle cingulate gyrus; DCG.R, right middle cingulate gyrus; ORBmed.L, left medial orbitofrontal cortex; ORBmed.R, right medial orbitofrontal cortex; SFGmed.L, left medial superior frontal gyrus; HIP.L, left hippocampus.

We also compared the BC of each region between MMD patients and HCs. Uncorrected analysis showed significantly reduced BC values among MMD patients in the bilateral medial orbitofrontal cortices (left: *P* = 0.001, right: *P* = 0.005), left medial superior frontal gyrus (*P* = 0.019) and left hippocampus (*P* = 0.026), and significantly increased BC in the bilateral middle cingulate gyri (left: *P* = 0.008, right: *P* = 0.006). Further, the regional BC values of the bilateral medial orbitofrontal cortices were still significantly lower in the MMD group after FDR correction for multiple comparisons (left: *P* = 0.045, right: *P* = 0.045) (Figure 3).

### Network Resilience

**FIGURE 4:**
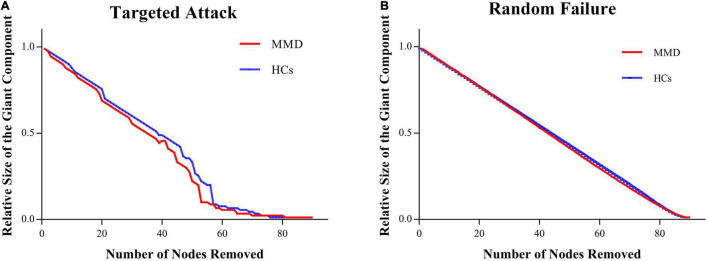
Between-group differences in network resilience. No difference is found between MMD and HCs in network resilience. **(A)** Targeted attack; **(B)** random failure.

The SCNs of MMD patients were as robust to targeted attack and random failure as those of HCs in both permutation analysis (all *P* > 0.05) and AUC analysis (all *P* > 0.05) (Figure 4).

## Discussion

Structural covariance networks including 90 brain regions differed markedly in topological properties between stroke-free MMD patients and well-matched controls at both the global and local levels. Further, many of these differences in SCN organization involved frontal and limbic regions implicated in higher-level cognition, possibly explaining the cognitive deficits observed in MMD even prior to major ischemic and hemorrhagic events. Nonetheless, the whole brain network of patients retained small-worldness and resilience to perturbation, suggesting that some of these changes serve as compensatory mechanisms.

### Altered Global Structural Covariance Network Measures

Networks with small-world topography exhibit both high efficiency for specialized information processing in local regions (functional segregation) and rapid integration of information over the entire network (functional integration) (Stam and Reijneveld, 2007; Rubinov and Sporns, 2010). However, several global measures of SCN (Lp, Cp, assortativity, local efficiency, and transitivity) were significantly reduced in the MMD group, indicating a suboptimal balance between functional integration and segmentation. The significant decreases in Cp and Lp among MMD patients are indicative of a more randomized SCN, in accord with previous functional network studies using rs-fMRI and DTI (Kazumata et al., 2016; Lei et al., 2020). Furthermore, the significantly decreased Cp indicates reduced interconnections among neighboring regions (nodes) and decreased local cliquishness (activity among clusters of local nodes) (Watts and Strogatz, 1998; Fleischer et al., 2019b). Transitivity is a variant of Cp with similar meaning, but is more robust as it is less influenced by nodes with small numbers of connections. This decreased transitivity provides further support for the notion that MMD results in aberrant cluster organization and less efficient modular processing by neighboring nodes (Newman and Park, 2003; Fleischer et al., 2019b). Indeed, local efficiency was also reduced significantly in MMD patients, further suggesting a reduced capacity for information transfer among neighboring nodes (Latora and Marchiori, 2001; Vragovic et al., 2005). These changes are also in line with decrease assortativity in the MMD group, which indicates reduced communication efficiency (Murakami et al., 2018). Collectively, lower Cp, transitivity, and local efficiency indicate reduced functional segregation and thus weaker local information processing in MMD.

However, we also found significantly reduced Lp in the MMD group, implying a shorter distance between all node pairs and thus enhanced information transfer capacity between remote regions of the brain (Watts and Strogatz, 1998). In addition, Lp is believed to reflect functional integration capacity from widespread regions, so functional integration of certain types of information may be enhanced, possible as a compensatory mechanism for reduced local information processing.

Previous studies of healthy volunteers have found associations between higher intelligence quotient and more integrated topological brain networks (Li et al., 2009; van den Heuvel et al., 2009), which have an advantage in difficult cognitive tasks (Kitzbichler et al., 2011; Crossley et al., 2013). However, MMD patients with reduced Lp suffer from cognitive impairments, even in the absence of major infarcts from strokes or hemorrhages. Similarly, patients with schizophrenia (Lo et al., 2015) or major depressive disorder (Li et al., 2017; Zhang et al., 2018) present increased functional integration, but suffer from cognitive impairments. One possible explanation is that increased functional integration is formed as a type of pathologic change or compensatory neuroplastic mechanism during disease process, which does nothing with higher-order cognitive processes. For schizophrenia and major depressive disorder, a more randomized brain network organization with higher integration has been proposed as a biomarker, which may serve as a kind of pathologic change (Lo et al., 2015; Li et al., 2017; Zhang et al., 2018). For MMD, we speculated that decreased Lp in MMD could reflect compensatory neuroplastic mechanisms against the inefficient information processing. Similarly, the brain can form new connections during recovery after ischemic attack (Lee et al., 2015), while enhancing the strength of conserved pathways or recruit other systems *via* neural circuit plasticity after trauma (Nishimura and Isa, 2009).

### Altered Regional Structural Covariance Network Measures

Regional BC values of bilateral medial orbitofrontal cortices were also significantly decreased in the MMD group after FDR correlation for multiple comparisons, which implies less efficient communications and longer paths of information transfer between these structures and other regions (Barthélemy, 2004). This finding is also consistent with a previous study reporting dysfunction of medial orbitofrontal cortex in adult MMD patients (Lei et al., 2017). Three cognitive processes may be especially disturbed by orbitofrontal cortex dysfunction. The orbitofrontal cortex contributes to reinforcing emotional stimuli during stimulus–reinforcer association learning, especially emotion-related learning (Rolls, 2019). Thus, stimulus-reinforcer association learning may be impaired in MMD. Second, damage to the orbitofrontal cortex limits cognitive flexibility for learning and adapting to changing reinforcement contingencies (Zald and Andreotti, 2010). Third, medial orbitofrontal cortex damage can interfere with optimal decision-making (Rolls and Grabenhorst, 2008). In addition, the medial orbitofrontal cortex is a key node of the default mode network (DMN) (Buckner et al., 2008) implicated in internally focused thoughts. Sakamoto et al. (2018) found that working memory and performance speed were inversely correlated with the degree of DMN disruption in MMD patients. Studies probing the associations between brain network reorganization involving the bilateral medial orbitofrontal cortices and specific cognitive impairments are needed.

The BC values of the left medial superior frontal gyrus and left hippocampus were also significantly reduced in the MMD group before FDR correction. The dorsomedial prefrontal cortex (approximating Brodmann Area 10), as a part of the medial superior frontal gyrus, is crucial for prospective memory (Burgess et al., 2003) and attention (Burgess et al., 2007), while the hippocampus is involved in encoding and storage of associative memory (Lisman et al., 2017). Furthermore, several studies have reported impaired memory and attention function in MMD patients (Fang et al., 2016; Shi et al., 2020). We also found increased BC in the bilateral middle cingulate gyri, indicating higher influences in network and shorter paths to reach other regions (Barthélemy, 2004), in accord with previous results (Kazumata et al., 2016; Lei et al., 2020). The middle cingulate gyrus is often considered a bridge connecting different regions (Dosenbach et al., 2007, 2008), so increased BC may again reflect compensatory network reorganization to maintain efficient information transfer in MMD patients. However, none of these network changes (except reduced connectivity of bilateral medial orbitofrontal cortices) was significant after FDR correlation. Thus, larger study cohorts are required to confirm these results and determine if these changes are heterogeneous across patient subgroups or consistent but small in magnitude among stroke-free MMD patients.

### Network Resilience

Surprisingly, the SCN of the MMD group was as resilient to targeted attack and random failure as that of the HC group. We suggested three potential reasons for the conserved robustness of otherwise reorganized SCNs in MMD. First, the SCN of the MMD group showed significantly lower clustering efficiency and lower characteristic path length, which indicates more random topology, and previous studies have shown that random networks can remain robust even after a large proportion of the nodes has been removed by random failure or targeted attack (Albert et al., 2000; Li et al., 2017; Zhang et al., 2018). The randomized feature of the SCN in MMD patients may thus enhance resilience. Second, all MMD patients enrolled were stroke-free, so there were no large ischemic lesions resulting in broad disruption of nodal connections. Third, false negatives are possible given the relatively small patient sample. Further studies are required to gauge the resilience of the SCN in MMD patients with and without major ischemic events.

### Applications of Structural Covariance Network

Structural covariance network analyses have been widely used in diseases, such as depression (Mak et al., 2016), multiple sclerosis (Fleischer et al., 2019a) and AD (Phillips et al., 2015), and physiological processes, such as maturation (Woodburn et al., 2021) and aging (Aboud et al., 2019). SCN provides a whole new approach to exploring the disruption and reorganization of complex brain networks. Moreover, several SCN studies found that the global efficiency increases as children grow and mature (Woodburn et al., 2021), and the degree of change/reorganization of the SCN is correlated with the severity of schizophrenia (Kim et al., 2020) and cognitive impairment in multiple sclerosis patients (Hawkins et al., 2020). Therefore, the SCN indices could be promising biomarkers in further studies. This study is the first attempt to explore the reorganization of SCN in MMD patients, providing a new perspective and some useful information on the mechanisms of cognitive impairments in MMD patients.

### Limitations

First, because this study only examined SCN changes based on measures of GM volume, findings could not reflect functional network reorganization or changes in WM tracts. SCN studies of MMD patient could provide additional information on the reorganization of brain network based on morphological features. Second, no cognitive examinations were conducted to establish correlations with network measures. Third, atlases with finer parcellation than the AAL atlas may provide more information about SCN reorganization in MMD patients.

## Conclusion

The SCNs of stroke-free ischemic MMD patients are reorganized at both the global and regional levels. Patients with MMD exhibit a less optimal and more randomized SCN compared with well-matched controls, while the nodal BC of the bilateral medial orbitofrontal cortices is severely reduced. However, the SCNs of MMD patients are as robust as those of HCs against targeted attack and random failure.

## Data Availability Statement

The datasets presented in this article are not readily available because of subjects’ privacy protection. Requests to access the datasets should be directed to YZ, yanzhang135@163.com.

## Ethics Statement

The studies involving human participants were reviewed and approved by the Ethics Committee of Beijing Tiantan Hospital, Capital Medical University. The patients/participants provided their written informed consent to participate in this study.

## Author Contributions

PW and YZ: conception and design. PW, WL, and HZ: acquisition of data. PW: analysis and interpretation of data and drafting the manuscript. XL, TY, DZ, and YZ: technical, administrative, and material support. YZ: study supervision and approving the final version of the manuscript on behalf of all authors. All authors critically revised and reviewed the submitted version of the manuscript.

## Conflict of Interest

The authors declare that the research was conducted in the absence of any commercial or financial relationships that could be construed as a potential conflict of interest.

## Publisher’s Note

All claims expressed in this article are solely those of the authors and do not necessarily represent those of their affiliated organizations, or those of the publisher, the editors and the reviewers. Any product that may be evaluated in this article, or claim that may be made by its manufacturer, is not guaranteed or endorsed by the publisher.
