# Supplementary material for: Reorganization of the Brain Structural Covariance Network in Ischemic Moyamoya Disease Revealed by Graph Theoretical Analysis
Source: Front Aging Neurosci. 2022 Jun 2;14:788661. doi: 10.3389/fnagi.2022.788661 (PMC9201423; doi:10.3389/fnagi.2022.788661)
Supplement: Supplementary file 1 [file Table_1.docx]

Supplementary Material

# Supplementary Table (1)

Supplementary Table 1. Regions schemes of AAL-90 atlas.

|  | **Abbreviation** | **AAL region** |  | **Abbreviation** | **AAL region** |
| --- | --- | --- | --- | --- | --- |
| 1 | AMYG.L | Left amygdala | 46 | IOG.R | Right inferior occipital gyrus |
| 2 | AMYG.R | Right amygdala | 47 | MOG.L | Left middle occipital gyrus |
| 3 | ANG.L | Left angular gyrus | 48 | MOG.R | Right middle occipital gyrus |
| 4 | ANG.R | Right angular gyrus | 49 | SOG.L | Left superior occipital gyrus |
| 5 | CAL.L | Left calcarine cortex | 50 | SOG.R | Right superior occipital gyrus |
| 6 | CAL.R | Right calcarine cortex | 51 | OLF.L | Left olfactory cortex |
| 7 | CAU.L | Left caudate nucleus | 52 | OLF.R | Right olfactory cortex |
| 8 | CAU.R | Right caudate nucleus | 53 | PAL.L | Left lenticular nucleus, pallidum |
| 9 | ACG.L | Left anterior cingulate gyrus | 54 | PAL.R | Right lenticular nucleus, pallidum |
| 10 | ACG.R | Right anterior cingulate gyrus | 55 | PCL.L | Left paracentral lobule |
| 11 | DCG.L | Left middle cingulate gyrus | 56 | PCL.R | Right paracentral lobule |
| 12 | DCG.R | Right middle cingulate gyrus | 57 | PHG.L | Left parahippocampal gyrus |
| 13 | PCG.L | Left posterior cingulate gyrus | 58 | PHG.R | Right parahippocampal gyrus |
| 14 | PCG.R | Right posterior cingulate gyrus | 59 | IPL.L | Left inferior parietal lobule |
| 15 | CUN.L | Left cuneus | 60 | IPL.R | Right inferior parietal lobule |
| 16 | CUN.R | Right cuneus | 61 | SPG.L | Left superior parietal gyrus |
| 17 | IFGoperc.L | Left inferior frontal gyrus (opercular) | 62 | SPG.R | Right superior parietal gyrus |
| 18 | IFGoperc.R | Right inferior frontal gyrus (opercular) | 63 | PoCG.L | Left postcentral gyrus |
| 19 | ORBinf.L | Left inferior frontal gyrus (inferior) | 64 | PoCG.R | Right postcentral gyrus |
| 20 | ORBinf.R | Right inferior frontal gyrus (inferior) | 65 | PreCG.L | Left precentral gyrus |
| 21 | IFGtriang.L | Left inferior frontal gyrus (triangular) | 66 | PreCG.R | Right precentral gyrus |
| 22 | IFGtriang.R | Right inferior frontal gyrus (triangular) | 67 | PCUN.L | Left precuneus |
| 23 | ORBmed.L | Left orbitofrontal cortex (medial) | 68 | PCUN.R | Right precuneus |
| 24 | ORBmed.R | Right orbitofrontal cortex (medial) | 69 | PUT.L | Left lenticular nucleus, putamen |
| 25 | MFG.L | Left middle frontal gyrus | 70 | PUT.R | Right lenticular nucleus, putamen |
| 26 | ORBmid.L | Left orbitofrontal cortex (middle) | 71 | REC.L | Left rectus gyrus |
| 27 | ORBmid.R | Right orbitofrontal cortex (middle) | 72 | REC.R | Right rectus gyrus |
| 28 | MFG.R | Right middle frontal gyrus | 73 | ROL.L | Left Rolandic operculum |
| 29 | SFGdor.L | Left superior frontal gyrus (dorsal) | 74 | ROL.R | Right Rolandic operculum |
| 30 | SFGmed.L | Left superior frontal gyrus (medial) | 75 | SMA.L | Left supplementary motor area |
| 31 | SFGmed.R | Right superior frontal gyrus (medial) | 76 | SMA.R | Right supplementary motor area |
| 32 | ORBsup.L | Left orbitofrontal cortex (superior) | 77 | SMG.L | Left supramarginal gyrus |
| 33 | ORBsup.R | Right orbitofrontal cortex (superior) | 78 | SMG.R | Right supramarginal gyrus |
| 34 | SFGdor.R | Right superior frontal gyrus (dorsal) | 79 | ITG.L | Left inferior temporal gyrus |
| 35 | FFG.L | Left fusiform gyrus | 80 | ITG.R | Right inferior temporal gyrus |
| 36 | FFG.R | Right fusiform gyrus | 81 | MTG.L | Left middle temporal gyrus |
| 37 | HES.L | Left Heschl gyrus | 82 | MTG.R | Right middle temporal gyrus |
| 38 | HES.R | Right Heschl gyrus | 83 | TPOmid.L | Left temporal pole (middle) gyrus |
| 39 | HIP.L | Left hippocampus | 84 | TPOmid.R | Right temporal pole (middle) gyrus |
| 40 | HIP.R | Right hippocampus | 85 | TPOsup.L | Left temporal pole (superior) gyrus |
| 41 | INS.L | Left insula | 86 | TPOsup.R | Right temporal pole (superior) gyrus |
| 42 | INS.R | Right insula | 87 | STG.L | Left superior temporal gyrus |
| 43 | LING.L | Left lingual gyrus | 88 | STG.R | Right superior temporal gyrus |
| 44 | LING.R | Right lingual gyrus | 89 | THA.L | Left thalamus |
| 45 | IOG.L | Left inferior occipital gyrus | 90 | THA.R | Right thalamus |
